# Fungemia by *Wickerhamomyces anomalus*—A Narrative Review

**DOI:** 10.3390/pathogens13030269

**Published:** 2024-03-21

**Authors:** Petros Ioannou, Stella Baliou, Diamantis P. Kofteridis

**Affiliations:** School of Medicine, University of Crete, 71003 Heraklion, Greece

**Keywords:** *Wickerhamomyces anomalus*, *Candida pelliculosa*, *Endomyces anomalus*, *Hansenula anomala*, *Pichia anomala*, fungemia

## Abstract

*Wickerhamomyces anomalus* has been previously classified as *Hansenula anomala*, *Pichia anomala*, and *Candida pelliculosa* and was recently reclassified in the genus *Wickerhamomyces* after phylogenetic analysis of its genetic sequence. An increasing number of reports of human infections by *W. anomalus* have emerged, suggesting that this microorganism is an emerging pathogen. The present review aimed to provide data on the epidemiology, antifungal resistance, clinical characteristics, treatment, and outcomes of fungemia by *W. anomalus* by extracting all the available information from published original reports in the literature. PubMed/Medline, Cochrane Library, and Scopus databases were searched for eligible articles reporting data on patients with this disease. In total, 36 studies involving 170 patients were included. The age of patients with fungemia by *W. anomalus* ranged from 0 to 89 years; the mean age was 22.8 years, the median age was 2.2 years, with more than 37 patients being less than one month old, and 54% (88 out of 163 patients) were male. Regarding patients’ history, 70.4% had a central venous catheter use (CVC), 28.7% were on total parenteral nutrition (TPN), 97% of neonates were hospitalized in the neonatal ICU (NICU), and 39.4% of the rest of the patients were hospitalized in the intensive care unit (ICU). Previous antimicrobial use was noted in 65.9% of patients. The most common identification method was the matrix-assisted laser desorption/ionization time-of-flight mass spectrometry (MALDI-TOF MS) in 34.1%, VITEK and VITEK 2 in 20.6%, and ID32 C in 15.3%. *W. anomalus* had minimal antifungal resistance to fluconazole, echinocandins, and amphotericin B, the most commonly used antifungals for treatment. Fever and sepsis were the most common clinical presentation noted in 95.8% and 86%, respectively. Overall mortality was 20% and was slightly higher in patients older than one year. Due to the rarity of this disease, future multicenter studies should be performed to adequately characterize patients’ characteristics, treatment, and outcomes, which will increase our understanding and allow drawing safer conclusions regarding optimal management.

## 1. Introduction

Fungi can cause several infections, such as superficial skin and oral, esophageal, and vulvovaginal infections, as well as invasive infections involving the lungs, the bloodstream, the heart, the liver, the spleen, and the central nervous system [1,2,3,4,5,6,7,8]. Importantly, they are important causes of morbidity and mortality in specific patient populations, such as patients with chronic obstructive pulmonary disease or lung cancer, patients in critical care departments, and patients with significant immunosuppression, such as those with organ transplantation, hematological malignancy, HIV-positive patients, and other individuals that are receiving immunosuppressive treatment [9,10,11,12,13,14]. From a clinical perspective, fungal pathogens are commonly divided into yeast, molds, and dimorphic fungi [15]. Yeasts are classically oval or round and form flat, smooth colonies, while they reproduce by budding [15]. *Candida* species are the most common yeast that causes disease in humans and may cause either superficial mucocutaneous disease or candidemia and invasive candidiasis [16]. More specifically, *C. albicans* is the most commonly identified species, while *C. glabrata*, *C. parapsilosis*, *C. tropicalis*, and *C. krusei* are also increasingly identified in clinical specimens of invasive candidiasis and candidemia [16]. However, other non-Candida yeasts can also cause invasive fungal disease in humans. Such species include *Trichosporon, Rhodotorula, Kodamaea,* and *Wickerhamomyces* species, and there are several reports of infections by these species in the literature [17,18,19,20].

*Wickerhamomyces anomalus* has been previously classified as *Hansenula anomala, Pichia anomala,* and *Candida pelliculosa* and was recently reclassified in the genus *Wickerhamomyces* after phylogenetic analysis of its genetic sequence [21]. This species can be found in the environment and, more specifically, on grapes in vineyards and in winery facilities, and has been used in wineries for the improvement of wine quality and production [22,23]. Additionally, toxins produced by this species could also be of medical use against specific microbial pathogens [24]. In that direction, *W. anomalus* has significant antifungal activity against several fungal pathogens in different hosts [25]. However, an increasing number of reports of human infections by *W. anomalus* have emerged, suggesting that this microorganism is an emerging pathogen. For example, there are reports of clonal outbreaks of invasive fungal disease, more commonly fungemia [26,27].

Given the rarity of fungemia by this pathogen, its epidemiology and clinical characteristics have not been adequately described. Indeed, only scarce case reports with literature reviews or small series can be found in the literature [28,29]. The present review aimed to provide data on the epidemiology, antifungal resistance, clinical characteristics, treatment, and outcomes of fungemia by *W. anomalus* by extracting all the available information from published original reports in the literature.

## 2. Materials and Methods

This narrative review extracted and collected data regarding *W. anomalus* fungemia in humans. The primary aim of this study was to provide information specifically regarding the mortality and the epidemiology of fungemia. A secondary outcome of the study was to provide data on (a) patients’ medical history, (b) antifungal resistance, (c) the patients’ clinical characteristics, and (d) their treatment. For the literature screening, PubMed/Medline, Cochrane Library, and Scopus databases were searched for eligible articles reporting “((Candida AND pelliculosa) OR (Wickerhamomyces OR Willia OR Endomyces OR Hansenula OR Pichia) AND anomal*) AND (fungemia OR fungaemia OR bloodstream)” until 12 February 2024. Inclusion criteria included studies providing original data, such as case reports, case series, and retrospective and prospective studies providing information about the epidemiology and outcomes of fungemia by *W. anomalus* in humans. Articles in a language different from English were excluded. Letters to the editor, reviews, and any other study not providing original information were excluded. Moreover, studies referring to animals and studies whose full text could not be found were excluded from further analysis. Finally, studies not providing sufficient data on the epidemiology and mortality of patients were also excluded. The references of the remaining articles were also searched to assess potential studies following the snowball procedure.

The extracted data included the year of publication, study type, and country; patients’ age and gender; patients’ relevant medical history (intensive care unit (ICU) or neonatal ICU (NICU) stay, total parenteral nutrition (TPN), presence of a central venous catheter (CVC) recent antibiotic use, antifungal prophylaxis, diagnosis of solid or hematologic malignancy, and chemotherapy); infection and relevant microbiology (infection site, method of microorganism identification, antifungal resistance, clinical characteristics, and complications); antifungal treatment administered; source control (for example, CVC removal), and outcomes (i.e., cure or death). Fungemia was defined as identifying the fungus in a patient’s blood cultures. The association of mortality with the infection and causal microbiology was reported according to the study authors.

Data are presented as numbers (%) for categorical variables and median (interquartile range, IQR) for continuous variables. Categorical data were analyzed using Fisher’s exact test. Continuous variables were compared using the Mann–Whitney U-test for non-normally distributed variables or the *t*-test for normally distributed variables. All tests were two-tailed, and a *p*-value equal to or lower than 0.05 was considered significant. A univariate linear regression analysis including all patients, irrespective of age, was conducted to identify factors associated with all-cause mortality. Statistics were calculated with GraphPad Prism 6.0 (GraphPad Software, Inc., San Diego, CA, USA).

## 3. Results

### 3.1. Included Studies’ Characteristics

**Figure 1:**
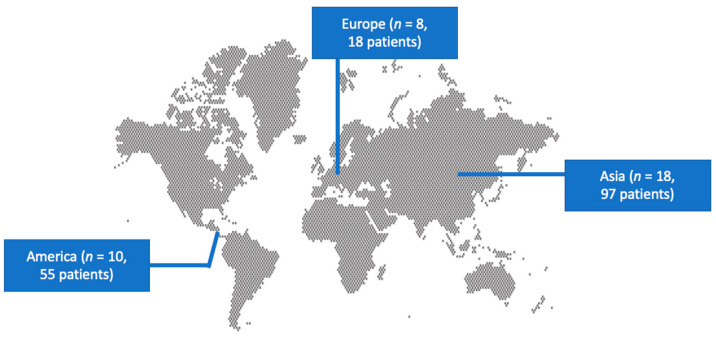
Geographical distribution of studies reporting fungemia by *W. anomalus* worldwide.

A total of 243 articles from PubMed and Scopus were screened. Finally, 36 met the present study’s inclusion criteria [19,26,27,28,29,30,31,32,33,34,35,36,37,38,39,40,41,42,43,44,45,46,47,48,49,50,51,52,53,54,55,56,57,58,59,60]. These 36 studies involved 170 patients in total. Among those studies, 18 were conducted in Asia, 10 in North and South America, and 8 in Europe. There were 16 case reports. Figure 1 shows the geographical distribution of *W. anomalus* fungemia cases worldwide. Table S1 shows the characteristics of the included studies.

### 3.2. Epidemiology of Fungemia by Wickerhamomyces anomalus

The age of patients with fungemia by *W. anomalus* ranged from 0 to 89 years; the mean age was 22.8 years, the median age was 2.2 years, with more than 37 patients being less than one month old, and 54% (88 out of 163 patients) were male. Regarding predisposing factors, 97% (65 out of 67 neonates and young babies with available data) were hospitalized in the NICU, and 89.2% (33 out of 37 patients with available data) had a low birth weight. Otherwise, 39.4% (39 out of 99 older patients with available data) were hospitalized in an ICU or pediatric ICU. Furthermore, 28.7% (39 out of 136 patients) were receiving TPN, 21.6% (32 out of 148) had hematologic malignancy, 12.2% (18 out of 148) had solid malignancy, 10.4% (14 out of 134) were receiving chemotherapy at the time of diagnosis, 5.2% (7 out of 134) were neutropenic at diagnosis, 70.4% (95 out of 135) had a CVC, 12.6% (18 out of 143) were on antifungal prophylaxis, 28.4% (31 out of 109, more commonly fluconazole) had a recent surgery, and 65.9% (87 out of 132) had recently received antibiotics. The patients’ characteristics from the studies that provide nonaggregated data can be seen in Table 1 and Table 2.

### 3.3. Microbiology, Identification, and Antifungal Resistance of Wickerhamomyces anomalus

Infection was polymicrobial in 16.4% (24 out of 146 patients), with blood cultures being positive for Gram-positive bacteria in 11 patients (5 for coagulase-negative staphylococci, and 2 for *Enterococcus* faecalis, for patients with known data), Gram-negative bacteria in 12 patients (3 patients for *Acinetobacter baumannii*, 2 patients for *Pseudomonas* spp., one patient for *Providencia rettgeri*, one patient for *Serratia marcescens*, 1 patient for *Klebsiella pneumoniae*, one patient for *Burkholderia cepacia*), and for *Candida* spp. in 4 patients.

The method for microorganism identification was not mentioned in 7.6% (13 out of 170 patients). The most common identification method was the matrix-assisted laser desorption/ionization time-of-flight mass spectrometry (MALDI-TOF MS) in 34.1% (58 patients), VITEK and VITEK 2 in 20.6% (35 patients), ID32 C in 15.3% (26 patients), PCR and DNA sequencing in 14.7% (25 patients), classic microbiology methods (morphology and biochemistry) in 14.7% (25 patients), and API 20 in 8.2% (14 patients).

Antifungal resistance was 26.3% (15 out of 57) to 5-fluocytosine, 18.5% (12 out of 65 strains) to voriconazole, 17.9% (10 out of 56) to itraconazole, 6.9% (6 out of 87) to fluconazole, 2.2% (2 out of 93) to amphotericin B, 0% (0 out of 22) to caspofungin, and 0% (0 out of 22) to micafungin. Table 1 and Table 2 show the antifungal resistance of *W. anomalus* in patients younger than 1 year old and older than 1 year old, respectively.

### 3.4. Clinical Characteristics of Fungemia by Wickerhamomyces anomalus

Infection was hospital-acquired in 78.5% (102 out of 130 patients), healthcare-associated in 1.5% (2 out of 130), and community-acquired in 1.5% (2 out of 130). The most common clinical presentation included fever in 95.8% (69 out of 72 patients) and sepsis in 86% (49 out of 57). Infective endocarditis was diagnosed in 1.6% (1 out of 64 patients), while endophthalmitis was not diagnosed in any of the 64 patients with relevant data.

### 3.5. Treatment and Outcomes of Fungemia by Wickerhamomyces anomalus

Treatment of patients with fungemia by *W. anomalus* is summarized in Table 1 and Table 2. Overall, and considering also the patients from studies with aggregated data, amphotericin B was used in 47.9% (68 out of 142 patients), fluconazole in 35.9% (51 patients), caspofungin in 4.9% (7 patients), micafungin in 4.9% (7 patients), voriconazole in 2.8% (4 patients), miconazole in 2.1% (3 patients), 5-flucytosine in 2.1% (3 patients), and itraconazole in 0.7% (1 patient). The median treatment among survivors was 20 days (range 14 to 49 days) for patients younger than one year and 17 days (range 8 to 42 days) for patients older than one year. Removal of a CVC was performed in 85.7% (30 out of 35 patients). Microbiological cure through the confirmation of sterilization of the blood was noted in 79.3% (23 out of 29 patients). Overall mortality was 20% (34 out of 170 patients), while in 14% (18 out of 129 patients), the death was directly attributed to the fungemia. Even though overall mortality was slightly higher in patients older than one year vs. those younger than one year (30.4% vs. 13.3%), this did not reach statistical significance (*p* = 0.0564).

### 3.6. Comparison of Patients with Fungemia by Wickerhamomyces anomalus Who Died with Those Who Survived

Table 1 and Table 2 compare patients with fungemia by *W. anomalus* who died with those who survived. In patients younger than one year who died, the hospitalization rate in the NICU at the time of diagnosis was lower than that in patients of the same age who survived. In patients older than one year, patients who died had higher age, were more likely to have received antibiotics in the three months preceding the diagnosis of fungemia, and were less likely to have removed the CVC during their hospitalization.

### 3.7. Statistical Analysis of Fungemia by Wickerhamomyces anomalus

In the univariate regression analysis, among the different parameters tested in patients with *W. anomalus* fungemia, higher age, receipt of antibiotics in the three months preceding the diagnosis of fungemia, and treatment with micafungin were positively associated with overall mortality (*p* = 0.0025, *p* = 0.0128, and *p* < 0.0001, respectively), while the removal of the CVC was negatively associated with overall mortality (*p* < 0.0001). A multivariate logistic regression did not identify any independent factors associated with the overall mortality of patients with fungemia by *W. anomalus*.

## 4. Discussion

This study describes the characteristics of patients with fungemia by *W. anomalus*. A significant proportion of patients were neonates or young babies; the rest were of varying ages, while most were male. Almost all cases were hospital-acquired, while hospitalization in the ICU, presence of a CVC, previous antibiotic use, TPN use, previous surgery, and malignancy were commonly described in patients’ history. Resistance to echinocandins and fluconazole was minimal. Fluconazole was the most commonly used antifungal for treatment. Mortality was high and was relatively lower in neonates and young babies than in older patients.

*Candida* species are the most common cause of invasive disease and fungemia by yeasts, while specific guidelines regarding their management exist [61]. However, other, less frequent yeast could also cause disease in humans, and their epidemiology, clinical characteristics, antifungal resistance, and optimal treatment are not fully described. For example, in a recent study from Spain, the epidemiology and antifungal resistance of yeasts causing fungemia was described [62]. Even though *Candida* species were the most commonly identified species, adding up to more than 95%, several other species, such as *Rhodotorula* spp., *Trichosporon* spp., *Magnesiomyces* spp., *Arxula* spp., and *Kodamaea* spp., were identified. *W. anomalus* is a relatively rare opportunistic yeast that may also cause infection in specific patient populations and under specific circumstances.

In the present study, two relatively independent groups of patients can be identified. The first one includes neonates and young babies, commonly hospitalized in the NICU and having a low birth weight. Indeed, neonates, especially those that have extremely low birth weight, are at an increased risk of acquiring invasive disease by yeasts, and more specifically, candidemia. It is estimated that up to 5% of these neonates may develop candidemia, leading to significant morbidity and mortality [63,64]. In a relevantly recent study including extremely low birth weight neonates, carbapenem use, TPN use, and the duration of hospitalization were identified in a multivariate regression analysis to be independently associated with a risk to develop candidemia in this patient population [65]. In a systematic review addressing the risk factors for the development of candidemia in neonates, 42 studies were eventually included, with 14 providing data for the meta-analysis [66]. The main risk factors for the development of candidemia in neonates were low birth weight, presence and duration of stay of CVCs, TPN use, mechanical ventilation, broad-spectrum antibiotics, and multiple antibiotics. Even though this study could not assess whether independent factors are associated with the development of fungemia by *W. anomalus*, both TPN use and low birth weight were very frequent in neonates and young babies, implying that they could be associated with a high risk for the development of this disease, as is the case with candidemia.

The second patient group includes non-neonates. These patients’ ages span from one year old to 89 years old. The use of antibiotics, CVC, and TPN is common in this patient population, while fungemia is almost exclusively hospital-acquired. However, in this patient population, contrary to the neonates and babies, a diagnosis of malignancy is very common, with many of these patients being treated with chemotherapy at the time of diagnosis of fungemia. Moreover, there was a trend for higher mortality in older patients. In a recent study by Poissy et al. that evaluated 192 patients with candidemia and 411 matched controls, several risk factors for the development of candidemia were identified in patients in the ICU and patients outside the ICU [67]. TPN use, acute kidney injury, previous septic shock, heart disease, and exposure to aminoglycosides were independent risk factors for the development of candidemia in the ICU population, while the presence of a CVC, TPN use, and exposure to glycopeptides or nitroimidazoles were identified as independent risk factors for the development of candidemia in patients outside the ICU [67]. Patients with malignancy may be at a higher risk for developing an invasive yeast infection, and more specifically, candidemia [68]. This has led to the introduction of antifungal prophylaxis in specific patient subgroups among immunosuppressed patients, including patients with malignancy, especially hematologic, who are receiving chemotherapy [69,70,71]. Antifungal prophylaxis may reduce the likelihood of the development of candidemia or fungemia by other yeasts in such patients. Interestingly, in the present review, a small proportion of patients were on antifungal prophylaxis; however, even though *W. anomalus* did not have significant antifungal resistance, breakthrough fungemia by this species occurred. This may be associated with an increased risk for the development of fungemia by these patients. On the other hand, in studies of breakthrough candidemia, the identified species, more commonly non-*albicans Candida* species, may be susceptible to the antifungal drug that had been used for prophylaxis [72]. This aligns with the studies in the present review, where the most commonly used antifungal drug was fluconazole—an antifungal to which *W. anomalus* was highly susceptible.

Regarding pathogen identification, the most commonly used method in the studies providing data for the present review was MALDI-TOF MS. Indeed, even though this method requires expensive equipment, it provides more reliable identification compared to classic microbiologic methods such as morphology and biochemical properties [73]. Indeed, MALDI-TOF MS has proven its ability to avoid misidentification even in complex cases, such as in the case of *C. auris,* where misidentification with other *Candida* species was common with other methods relying on biochemical properties of the isolated strains [74,75,76].

Regarding antifungal resistance, *W. anomalus* was sensitive to most antifungals tested, including fluconazole and echinocandins. This is of particular importance since the clinician caring for a patient with fungemia by *W. anomalus* will be asked to treat a patient having, at first empirically, only the information that yeasts are isolated in the blood culture while identification is pending. Thus, since *Candida* spp. is the most commonly isolated yeast, it is reasonable that clinicians empirically treat patients based on the guidelines about invasive candidiasis. These guidelines currently suggest empirical treatment with an echinocandin, and, based on the data from the current review, this is expected to cover *W. anomalus* adequately [61]. Upon receipt of antifungal susceptibility data, de-escalation to fluconazole could be an adequate option if susceptibility is confirmed. The current review identified fluconazole and amphotericin B as the most commonly used antifungals for treating this fungus. Notably, many studies included in the present review were published before echinocandins were available; thus, these drugs could not have been used then. Indeed, the introduction of echinocandins has led to a reduction in amphotericin B use. At the same time, their efficacy seems to be at least equal to, or even higher than, that of amphotericin B, at least when data from patients with invasive candidiasis and candidemia are evaluated [77].

The clinical presentation of fungemia by *W. anomalus* most commonly included fever and sepsis, while no patient was diagnosed with endophthalmitis in the studies evaluated in the present review. Notably, in candidemia, the rate of endophthalmitis was identified in a recent systematic review to be between 1% and 4%, depending on the geographic region where candidemia occurred [78]. The patients in the current review are relatively few, and more data are required to draw safe conclusions; however, it could be that this fungus does not have a significant tendency to cause endophthalmitis.

Mortality in the current review was relatively high, and there was a trend for higher mortality in older patients. In patients older than one year, the mortality rate was 30%, which is comparable to the 30-day mortality rate of patients with candidemia, which was about 37% in other studies [79]. Even though the statistical analysis in the present study did not identify any factors independently associated with mortality, higher age, previous treatment with antibiotics, and treatment with micafungin were associated with higher mortality. Since these associations were not confirmed with the multivariate regression analysis, they could be just epiphenomena. In other studies with patients with candidemia, higher age was associated with higher mortality, among other factors [80]. Importantly, the removal of CVC in patients with *W. anomalus* fungemia in the current review was associated with reduced mortality. This is a well-known concept that is related with adequate source control and has been recognized as an essential parameter in the treatment of patients with candidemia as well. For example, there are studies suggesting that a delay in the removal of a CVC may lead to increased mortality in patients with candidemia [81].

The present study has some notable limitations. First, the data are mainly derived from a relatively small number of case reports and case series. Thus, the quality of evidence is relatively low. Larger studies are required to allow for drawing safer conclusions regarding the clinical presentation, the treatment, and the outcomes of patients suffering from this infection. Since fungemia by *W. anomalus* is a relatively rare condition, conducting such a study would require enormous efforts from multiple centers for a long time to allow enrollment of an adequate number of patients. Finally, in the discussion section, a comparison of cases of fungemia by *W. anomalus* with candidemia was made. These two conditions may not be directly comparable since these two yeasts differ; however, we chose to compare them since candidemia is the most common fungemia by yeast.

## 5. Conclusions

Fungemia by *W. anomalus* is a relatively rare yeast infection with significant mortality. Patients typically include neonates and babies receiving intensive care, more commonly in the NICU, or older patients who commonly suffer from immunosuppression due to malignancy and receipt of chemotherapy. In both patient populations, TPN and CVC use are very common. Accurate pathogen identification often relies on MALDI-TOF MS or PCR and sequencing. The fungus is usually susceptible to echinocandins and fluconazole, which could be currently considered the mainstay of treatment. Source control, such as the removal of contaminated CVCs, is paramount. Due to the rarity of this disease, future multicenter studies should be performed to adequately characterize patients’ characteristics, treatment, and outcomes, which will increase our understanding and allow drawing safer conclusions regarding optimal management.

## Figures and Tables

**Table 1:** Characteristics of patients younger than 12 months from studies providing nonaggregated data regarding the outcome of *Wickerhamomyces anomalus* infection.

Characteristic	All Patients Younger than 12 Months (*n* = 45) *	Survived (*n* = 39)	Died (*n* = 6)	*p*-Value
Age, years, median (IQR)	0 (0–0.08)	0.04 (0–0.08)	0 (0–0.06)	0.3799
Male gender, *n* (%)	18/39 (46.2)	17/33 (51.5)	1 (16.7)	0.1897
Predisposing factors				
Previously on antibiotics, *n* (%)	33/41 (80.5)	28/36 (77.8)	5/5 (100)	0.5632
In NICU, *n* (%)	38/40 (95)	34/35 (97.1)	4/5 (80)	0.0006
Low birth weight, *n* (%)	33/37 (89.2)	29/33 (87.9)	4/4 (100)	1
Post-surgery, *n* (%)	6/30 (20)	3/24 (12.5)	3 (50)	0.0754
Post-cardiac surgery, *n* (%)	4/31 (12.9)	2/25 (8)	2 (33.3)	0.1594
Total parenteral nutrition, *n* (%)	14/26 (50)	11/21 (52.4)	2/5 (40)	1
CVC, *n* (%)	14/15 (93.3)	9/10 (90)	5/5 (100)	1
Antifungal prophylaxis, *n* (%)	2/21 (9.5)	2/16 (12.5)	0/5 (0)	1
Antifungal resistance, *n* (%)				
Amphotericin B, *n* (%)	2/38 (5.3)	2/33 (6.1)	0/5 (0)	1
Fluconazole, *n* (%)	4/37 (10.8)	2/32 (6.3)	2/5 (20)	0.08
Voriconazole, *n* (%)	8/35 (22.9)	7/31 (22.6)	1/4 (25)	1
Caspofungin, *n* (%)	0/8 (0)	0/6 (0)	0/2 (0)	1
Micafungin, *n* (%)	0/10 (0)	0/9 (0)	0/1 (0)	1
Hospital-acquired infection, *n* (%)	42/43 (97.7)	36/37 (97.3)	6 (100)	1
Clinical characteristics				
Polymicrobial infection, *n* (%)	6 (13.3)	4 (10.3)	2 (33.3)	0.2672
Fever, *n* (%)	21/22 (95.5)	17/17 (100)	4/5 (80)	0.2273
Sepsis, *n* (%)	23/25 (92)	20/21 (95.2)	3/4 (75)	0.3000
Endophthalmitis, *n* (%)	0/25 (0)	0/20 (0)	0/5 (0)	1
Endocarditis, *n* (%)	1/25 (4)	0/20 (0)	1/5 (20)	0.2000
Treatment				
Amphotericin B, *n* (%)	21/44 (47.7)	17/38 (44.7)	4 (66.7)	0.6628
Fluconazole, *n* (%)	27/44 (61.4)	24/38 (63.2)	3 (50)	0.6619
Voriconazole, *n* (%)	1/44 (2.3)	1/38 (2.6)	0 (0)	1
Caspofungin, *n* (%)	1/44 (2.3)	0/38 (0)	1 (16.7)	0.1364
No treatment, *n* (%)	1/44 (2.3)	1/38 (2.6)	0 (0)	1
CVC removal, *n* (%)	6/8 (75)	5/5 (100)	1/3 (33.3)	0.1071
Outcomes				
Deaths due to infection, *n* (%)	2 (4.4)	NA	NA	NA
Deaths overall, *n* (%)	6 (13.3)	NA	NA	NA

CVC: central venous catheter; IQR: interquartile range; NA: not applicable; NICU: neonatal intensive care unit; *: data are among the number of patients mentioned on top unless otherwise described.

**Table 2:** Characteristics of patients older than 12 months from studies providing nonaggregated data regarding the outcome of *Wickerhamomyces anomalus* infection.

Characteristic	All Patients Older than 12 Months (*n* = 56) *	Survived (*n* = 39)	Died (*n* = 17)	*p*-Value
Age, years, median (IQR)	43.5 (17.3–62.5)	36 (17–56)	61 (31–72.5)	0.0449
Male gender, *n* (%)	29/55 (52.7)	20/38 (52.6)	9 (52.9)	1
Predisposing factors				
Previously on antibiotics, *n* (%)	29/39 (74.4)	17/27 (63)	12/12 (100)	0.0172
In ICU, *n* (%)	21/54 (38.9)	12/38 (31.6)	9/16 (56.3)	0.1280
Post-surgery, *n* (%)	14/35 (40)	10/26 (38.5)	4/9 (44.4)	1
Total parenteral nutrition, *n* (%)	25/41 (61)	18/30 (60)	7/11 (63.6)	1
CVC, *n* (%)	40/51 (78.4)	30/38 (78.9)	10/13 (76.9)	1
Hematologic malignancy, *n* (%)	10/54 (18.5)	9/38 (23.7)	1/16 (6.3)	0.2495
Solid malignancy, *n* (%)	18/54 (33.3)	13/38 (34.2)	5/16 (31.3)	1
Chemotherapy	13/41 (31.7)	12/29 (41.4)	1/12 (8.3)	0.0642
Antifungal prophylaxis, *n* (%)	4/53 (7.5)	1/37 (2.7)	3/16 (18.8)	0.0770
Antifungal resistance, *n* (%)				
Amphotericin B, *n* (%)	0/38 (0)	0/26 (0)	0/12 (0)	1
Fluconazole, *n* (%)	2/33 (6.1)	2/21 (6.3)	0/12 (0)	0.5227
Voriconazole, *n* (%)	4/30 (22.9)	3/18 (16.7)	1/12 (8.3)	0.6315
Caspofungin, *n* (%)	0/14 (0)	0/9 (0)	0/5 (0)	1
Micafungin, *n* (%)	0/12 (0)	0/7 (0)	0/5 (0)	1
Healthcare-associated infection **, *n* (%)	2/45 (4.4)	2/32 (6.3)	0/13 (0)	1
Hospital-acquired infection, *n* (%)	41/45 (91.1)	28/32 (87.5)	13/13 (100)	0.3076
Clinical characteristics				
Polymicrobial infection, *n* (%)	17 (30.4)	12 (30.8)	5 (29.4)	1
Fever, *n* (%)	30/32 (93.8)	22/24 (91.7)	8/8 (100)	1
Sepsis, *n* (%)	9/15 (60)	6/12 (50)	3/3 (100)	0.2286
Endophthalmitis, *n* (%)	0/22 (0)	0/18 (0)	0/4 (0)	1
Endocarditis, *n* (%)	0/22 (4)	0/18 (0)	0/4 (20)	1
Treatment				
Amphotericin B, *n* (%)	15 (26.8)	11 (28.2)	4 (23.2)	1
Fluconazole, *n* (%)	24 (42.9)	16 (41)	8 (47.1)	0.7722
Voriconazole, *n* (%)	3 (5.4)	1 (2.6)	2 (11.8)	0.2159
Micafungin, *n* (%)	7 (12.5)	3 (7.7)	4 (23.5)	0.1820
Caspofungin, *n* (%)	6 (10.7)	0 (0)	0 (0)	1
No treatment, *n* (%)	6 (10.7)	3 (7.7)	3 (17.6)	0.3540
CVC removal, *n* (%)	23/26 (88.5)	21/21 (100)	2/5 (40)	0.0038
Outcomes				
Deaths due to infection, *n* (%)	12 (21.4)	NA	NA	NA
Deaths overall, *n* (%)	17 (30.4)	NA	NA	NA

CVC: central venous catheter; ICU: intensive care unit; IQR: interquartile range; NA: not applicable; *: data are among the number of patients mentioned on top unless otherwise described; **: healthcare-associated infection herein includes cases associated with recent contact with the healthcare system but does not include cases of hospital-acquired infection that are reported separately.

## Data Availability

The data presented in this study are available on request from the corresponding author.

## Supplementary Materials

The following supporting information can be downloaded at: https://www.mdpi.com/article/10.3390/pathogens13030269/s1, Table S1: Characteristics of included studies.
