# Supplementary material for: Fungemia by Wickerhamomyces anomalus—A Narrative Review
Source: Pathogens. 2024 Mar 21;13(3):269. doi: 10.3390/pathogens13030269 (PMC10974086; doi:10.3390/pathogens13030269)
Supplement: Supplementary file 1 [file pathogens-13-00269-s001.zip › pathogens-2911165-supplementary.pdf]

Table S1. Characteristics of included studies

Author and year	Number of patients	Gender	Age (years)	Treatment (%)	Mortality (%)
Baron et al, 1988 [30]	1	Male	34	Amphotericin B 1 (100)	0 (0)
Klein et al, 1988 [31]	2	2 males	55, 59	Amphotericin B 2 (100)	0 (0)
Muñoz et al, 1989 [32]	1	Male	51	Amphotericin B 1 (100)	0 (0)
Salesa et al, 1991 [33]	1	Male	28	No antifungal 1 (100)	0 (0)
Neumeister et al, 1992 [34]	1	Male	65	Fluconazole 1 (100)	1 (100)
Hirasaki et al, 1992 [35]	1	Male	63	Fluconazole 1 (100)	0 (0)
Sekhon et al, 1992 [36]	1	Male	0.5	Amphotericin B 1 (100)<br>5-flucytosine 1 (100)	0 (0)
Yamada et al, 1995 [37]	4	4 males	0.033, 0.33, 2, 8	Fluconazole 3 (75)<br>Miconazole 1 (25)<br>5-flucytosine 1 (25)	1 (25)
Kunová et al, 1996 [38]	1	Female	46	Amphotericin B 1 (100)	0 (0)
Krcmery et al, 1998 [39]	1	Female	NR	Amphotericin B 1 (100)	0 (0)

Wong et al, 2000 [40]	1	Female	0	Amphotericin B 1 (100)	1 (1000)
Kalenic et al, 2001 [41]	8	5 males<br>3 females	22, 31, 32, 37, 41, 48, 58, 61	Fluconazole 5 (62.5)<br>Miconazole 2 (25)	3 (37.5)
Bakir et al, 2004 [42]	5	4 males<br>1 female	0, 0.92, 1.5, 8, 14	Amphotericin B 5 (100)<br>No antifungal 3 (37.5)	1 (20)
Paula et al, 2006 [43]	2	2 females	0, 0	Amphotericin B 2 (100)<br>Fluconazole 2 (100)<br>5-flucytosine 1 (50)	2 (100)
Krcmery et al, 2009 [44]	3	NR	0.08, 0.67, 8	Amphotericin B 3 (100)	0 (0)
Kalkanci et al, 2010 [45]	4	NR	0, 0, 0, 0	Amphotericin B 3 (75)<br>Fluconazole 2 (50)<br>Voriconazole 1 (25)<br>No antifungal 1 (25)	0 (0)
DeHaan et al, 2011 [46]	1	Male	70	Caspofungin 1 (100)	0 (0)
da Silva et al, 2013 [27]	5	2 males<br>3 females	0, 0, 0, 0, 0	Amphotericin B 2 (40)<br>Fluconazole 5 (100)	0 (0)

Chan et al, 2013 [47]	1	Male	21	Micafungin 1 (100)	0 (0)
Lin et al, 2013 [60]	6	3 males<br>3 females	0, 0, 0, 0, 0, 0	Amphotericin B 6 (100)<br>Fluconazole 1 (16.7)	1 (16.7)
Taj-Aldeen et al, 2014 [48]	1	Female	1.5	Amphotericin B 1 (100)	1 (100)
Otağ et al, 2015 [49]	2	2 females	0.14, 1.08	Caspofungin 1 (50)<br>No antifungal 1 (50)	1 (50)
Yılmaz-Semerci et al, 2017 [50]	1	Male	0.08	Fluconazole 1 (100)	0 (0)
Fernández-Ruiz et al, 2017 [51]	2	1 male<br>1 female	56, 75	Amphotericin B 1 (50)<br>Fluconazole 2 (100)	0 (0)
Jung et al, 2018 [52]	11	5 males<br>6 females	2, 6, 6, 17, 18, 47, 50, 52, 76, 77, 78	Fluconazole 2 (18.2)<br>Caspofungin 5 (45.5)<br>Micafungin 6 (54.5)	4 (36.4)
Dutra et al, 2020 [53]	1	Female	2.16	Amphotericin B 1 (100)<br>Fluconazole 1 (100)	1 (100)
Mehta et al, 2020 [54]	1	Male	36	Fluconazole 1 (100)	0 (0)
Cai et al, 2021 [29]	2	1 male	0, 0	Fluconazole 1 (50)	0 (0)

		1 female		NR 1 (50)	
Zhang et al, 2021 [19]	13	6 males<br>7 females	29, 33, 39, 41, 47, 60, 61, 64, 66, 69, 70, 75, 89	Amphotericin B 1 (7.7)<br>Fluconazole 9 (69.2)<br>Voriconazole 3 (23.1)<br>Itraconazole 1 (7.7)<br>No antifungal 1 (7.7)	5 (38.5)
Yang et al, 2021 [26]	14	8 males<br>6 females	0.04, 0.04, 0.04, 0.04, 0.06, 0.06, 0.06, 0.06, 0.07, 0.08, 0.08, 0.1, 0.1, 0.12	Fluconazole 14 (100)	0 (0)
Shubham et al, 2021 [28]	2	1 male<br>1 female	0, 0	Amphotericin B 2 (100)	0 (0)
Aboutalebian et al, 2023 [55]	1	male	5	Amphotericin B 1 (100)	1 (100)
Sakai et al, 2024 [56]	1	male	84	Amphotericin B 1 (100)	0 (0)
Thuler et al, 1997 [57]	24	11 males<br>13 females	11 (median)	Amphotericin B 16 (66.7)<br>No antifungal 8 (33.3)	0 (0)
Pasqualotto et al, 2005 [58]	17	9 males<br>8 females	1.1 (median)	Amphotericin B 15 (88.2)	7 (41.2)

				No antifungal 2 (11.7)	
Kaur et al, 2020 [59]	27	21 males<br>6 females	0.04 (median)	NR	4 (14.8)

NR: not reported
